# Simulation and Experimental Study on Anti-reflection Characteristics of Nano-patterned Si Structures for Si Quantum Dot-Based Light-Emitting Devices

**DOI:** 10.1186/s11671-016-1530-6

**Published:** 2016-06-29

**Authors:** Wenyi Shao, Peng Lu, Wei Li, Jun Xu, Ling Xu, Kunji Chen

**Affiliations:** National Laboratory of Solid State Microstructures and Jiangsu Provincial Key Laboratory of Advanced Photonic and Electronic Materials, School of Electronic Science and Engineering and Collaborative Innovation Center of Advanced Microstructures, Nanjing University, Nanjing, China

**Keywords:** Si nano-patterned structures, Anti-reflection, Photoluminescence

## Abstract

Surface-textured structure is currently an interesting topic since it can efficiently reduce the optical losses in advanced optoelectronic devices via light management. In this work, we built a model in finite-difference time-domain (FDTD) solutions by setting the simulation parameters based on the morphology of the Si nanostructures and compared with the experimental results in order to study the anti-reflection behaviors of the present nano-patterned structures. It is found that the reflectance is gradually reduced by increasing the depth of Si nanostructures which is in well agreement with the experimental observations. The reflectance can be lower than 10 % in the light range from 400 to 850 nm for Si nano-patterned structures with a depth of 150 nm despite the quite low aspect ratio, which can be understood as the formation of gradually changed index layer and the scattering effect of Si nano-patterned structures. By depositing the Si quantum dots/SiO_2_ multilayers on nano-patterned Si substrate, the reflectance can be further suppressed and the luminescence intensity centered at 820 nm from Si quantum dots is enhanced by 6.6-fold compared with that of flat one, which can be attributed to the improved light extraction efficiency. However, the further etch time causes the reduction of luminescence intensity from Si quantum dots which may ascribe to the serious surface recombination of carriers.

## Background

Crystalline Si plays a crucial role in today’s microelectronics industry, but it is hard to be utilized in optoelectronic devices because of its indirect band gap. The indirect band gap can cause low light-emitting efficiency and low absorption coefficient, which impede the improvement of device properties [1, 2]. Compared with the bulk Si materials, Si quantum dot (Si QD)-based materials exhibit the efficient light emission ability due to the enhanced recombination efficiency of electron-hole pairs in quantum-confined system [3]. So far, Si QD-based materials such as Si QDs/SiO_2_, Si QDs/SiN_x_, and Si QDs/SiC_x_ structures have been fabricated, and the photoluminescence and photovoltaic properties have been studied extensively [4–6]. However, the large index difference between Si and Si-based dielectric materials (SiO_2_, SiN_x_, or SiC_x_) causes strong light reflection which generates the optical losses in Si-based photo-electronic devices. For example, it was reported that in the Al_2_O_3_/ZnS:Mn system, though the index difference is relatively small (1.6 vs. 2.5), only 7.4 % light can be extracted from the front surface of the device [7]. It is reasonable to imagine that the situation will become more serious in Si/SiO_2_ system.

Recently, surface-textured structure using the nano-sphere lithography technique is currently an interesting topic since it can efficiently reduce the optical losses in advanced optoelectronic devices such as light-emitting diodes and solar cells via light management [8–11]. For example, Hsieh et al. fabricated textured GaN-based light-emitting diodes (LEDs) by nano-sphere lithography technique which obviously improved the device performance [9]. They have achieved an average of 38.5 % efficiency improvement of the textured p-GaN LED over the conventional device at a bias current of 20 mA. In our previous work, nano-sphere lithography technique was used to prepare nano-patterned Si structures. It was demonstrated that nano-patterned structures can improve the property of light-emitting devices as well as the heterojunction Si thin film solar cells [12–14]. In order to further understand the light management behaviors of Si-based nanostructures and to improve the device performance, it is necessary to systematically study the anti-reflection characteristics as a function of the parameters of the formed nanostructures. In the present work, both the simulation and experimental characterization is used to study the anti-reflection characteristics of periodically Si nano-patterned structures prepared by nano-sphere lithography technique. Based on the structural parameters extracted from atomic force microscopy (AFM) results, we built a model in finite-difference time-domain (FDTD) solutions. The influences of structural parameters on the optical properties of Si-nano-patterned structures were simulated and compared with the experimental results. Furthermore, we deposited Si quantum dots/SiO_2_ multilayers on the Si nano-patterned structures, and the luminescence intensity is enhanced by 6.6-folds.

## Methods

**Fig. 1:**
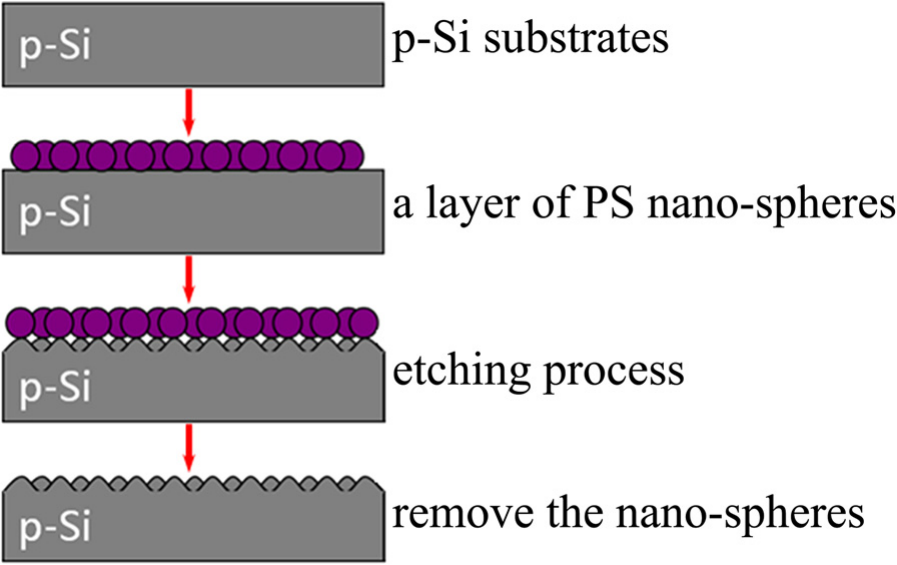
Fabrication process of nano-patterned Si structures

Nano-sphere lithography technique is used to fabricate Si nano-patterned structures. Before the fabrication process, p-Si substrates are cleaned by standard RCA process. As shown in Fig. 1, a small amount of the suspension of polystyrene (PS) nano-spheres and ethyl alcohol was first applied onto the surface of a clean silicon wafer. Subsequently, the silicon wafer was immersed into the deionized water to transfer the nano-spheres to the surface of water. Dodecyl sodium sulfate solution was added to control the surface tension. After setting quietly for 10 min, a monolayer of nano-sphere array with closed packed structures can be self-assembly formed on the water surface. Then, the monolayer was transferred to the surface of 1 cm × 1 cm flat Si substrate. The diameter of PS nano-spheres is about 300 nm, and the size of the monolayer on the water surface is about 3 cm × 5 cm. The Si substrates with coating by the PS nano-sphere array are then etched in reactive ion etching system by using PS nano-sphere monolayer as a mask [15]. During the etching process, CF_4_ is used as etching gas and the etching power is kept at 50 W. By controlling the etching time, the nano-patterned structures with various depths can be obtained. Finally, the PS nano-spheres on the Si substrates are removed by using ultrasonic cleaning technique in tetrahydrofuran [16].

**Fig. 2:**
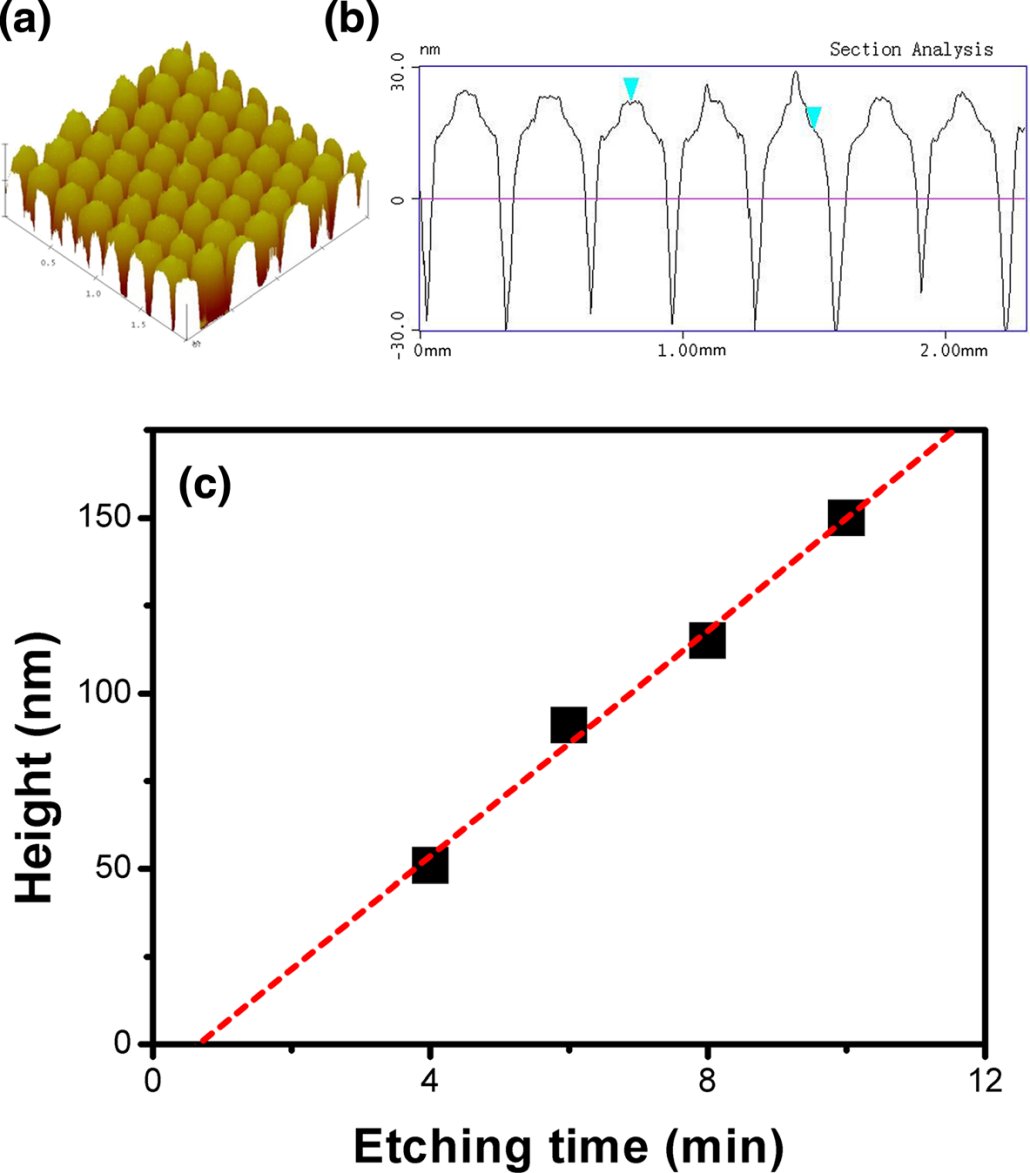
**a** AFM image and **b** cross-section picture of nano-patterned Si structures. **c** Linear fit of the relationship between etching time and height of Si nanostructures

The surface morphology of Si nano-patterned structures is characterized by AFM microscopy. Figure 2a shows the top morphology of the formed Si nano-patterned structures prepared by using 300 nm nano-spheres. A periodic and uniform structure can be identified in the AFM image. Figure 2b is the cross-section picture, which shows that the periodicity of nano-patterned structures is 300 nm and the depth is about 51 nm so that the aspect ratio defined as the ratio of depth (*H*) to diameter (*D*) (*H*/*D*) is less than unity. Figure 2c shows the relationship between the depth of nano-patterned structures and the etching time. The linear relationship indicates that the surface morphology of nano-patterned structures can be well controlled by changing the etching parameters. The reflection spectra of the prepared Si nano-patterned structures were measured by Shimadzu UV-3600 spectrophotometer in the range of 400–800 nm.

Furthermore, Si QDs/SiO_2_ multilayers (MLs) are fabricated on the Si nano-patterned structures by annealing amorphous Si/SiO_2_ stacked layers at 1000 °C for 1 h. The average size of Si QDs is about 2 nm, and the thickness of SiO_2_ layers is 2 nm too. The detailed fabrication process and conditions of Si QDs/SiO_2_ multilayers and TEM images can be found elsewhere in [16]. Room temperature photoluminescence measurements are performed by excited He–Cd laser with wavelength of 325 nm. As a reference, the Si QDs/SiO_2_ MLs are also prepared on a flat Si substrate for comparison.

## Results and Discussion

**Fig. 3:**
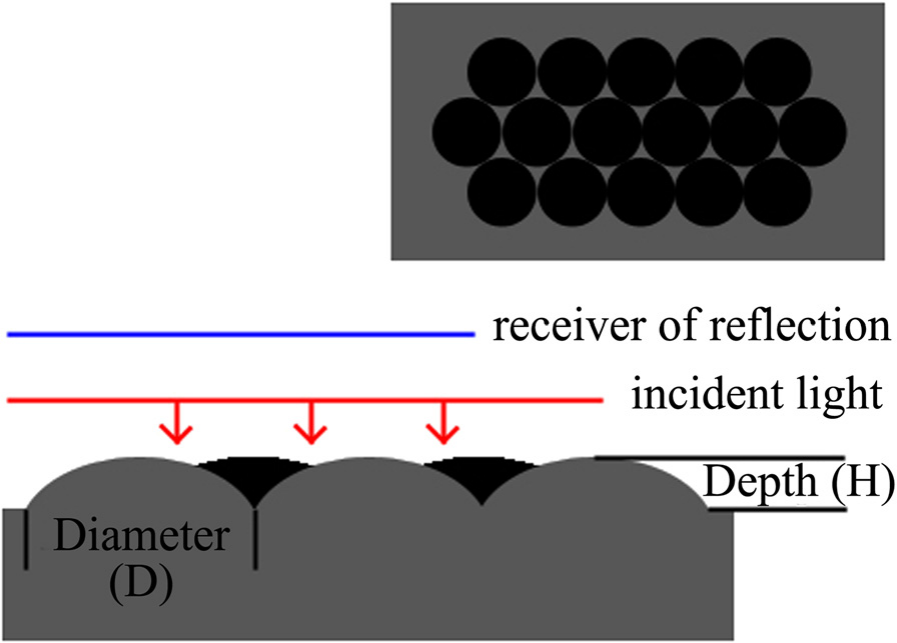
Schematic diagram of structural model for simulation

The optical reflection behaviors of formed nano-patterned structures are studied both theoretically and experimentally. Finite-difference time-domain (FDTD) solutions are used to simulate the reflection spectra of Si nano-patterned structures with variety morphologies by resolving 3D Maxwell’s equations. As shown in Fig. 3, the structural model with closed packed paraboloid array is built. Our model structure is more close to the experimental results by using a nano-sphere lithography technique as revealed by the AFM observations, which is different from the previous work by using a structure of poly-Si film textured by the hemispherical nanopit array [17]. During the simulation, the incident light is normally to the front surface of nano-patterned structures and the far-field reflection is calculated at the top situation.

**Fig. 4:**
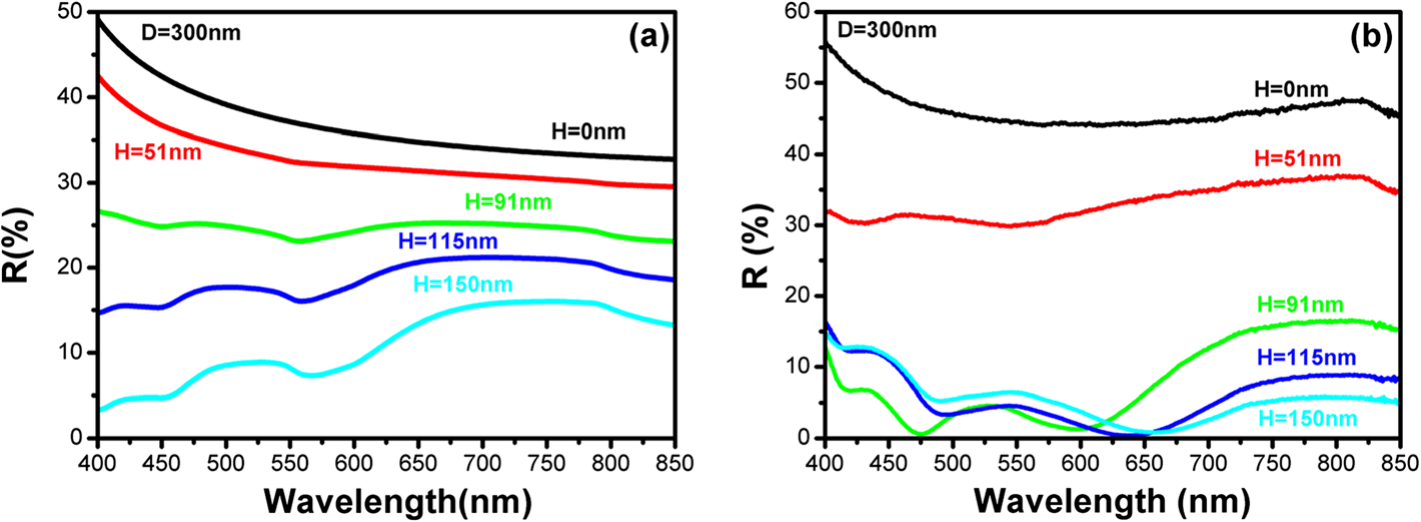
**a** Simulated reflection spectra and **b** experimental reflection spectra of samples with same diameter (*D* = 300 nm) and different depths (from 0 to 150 nm)

The influence of depth (*H*) and diameter (*D*) on the reflection behaviors of Si nano-patterned structures are simulated and studied. Figure 4a is the simulated reflectance spectra of Si nano-patterned structures with various depths by keeping the diameter (*D*) at 300 nm. It is found that the reflectance is quite high for flat Si substrates, which exceeds 35 % in the whole spectral range (400–850 nm). By forming the Si nano-patterned structures, the reflectance is gradually reduced by increasing the etching depth. The simulation results indicate that the broad-band anti-reflection ability of the Si nanostructures can be obtained by controlling the parameters of the formed Si. The reflectance can be lower than 10 % in the visible light range for Si nano-patterned structures with depth of 150 nm even if the aspect ratio of present Si nanostructures was quite small. The experimental data of the reflection from 400 to 850 nm are given in Fig. 4b to compare with the simulation results. It can be proved in both experimental and simulation results that the reflection will decrease as the increasing of depth. It is worth noting that the reflectance in the experiment decreases much faster as the depth increases. One of the possible reasons is that the surface topography will deviate from the paraboloid model when the depth increases. Since we treated the structures with the smooth surface, but the real surface after RIE etching is still quite rough. It was reported that the compound random nanostructures can significantly reduce the reflection compared with that of simple nanostructures [18].

The etched deep hole between the Si nano-patterned structures may also contributed to the anti-reflection behaviors. Usually, chemical etching methods were used to fabricate nano-hole structures [19]. The etching rate is very fast, and the depth of nano-holes usually exceeds 200 nm and the aspect ratio of nano-holes is much larger than 1. In our case, the aspect ratio of height/diameter is no more than 0.5 and the hole is not so deep to influence the observed anti-reflection characteristics strongly, though we cannot completely rule out of the contribution from nano-hole structures.

**Fig. 5:**
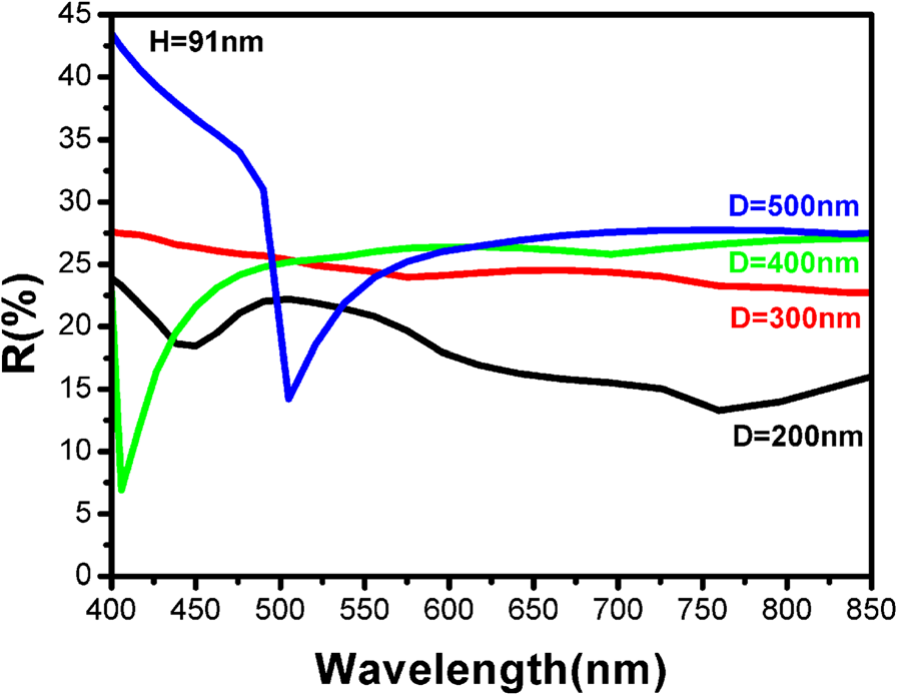
Simulated reflection spectra of samples with same height (*H* = 91 nm) and different diameters (from 200 to 500 nm)

Figure 5 shows the simulation results of reflection spectra of Si nano-patterned structures with various diameters (*D*) by keeping the depth at 91 nm. When the aspect ratio is high (*D* = 200 nm, *H* = 91 nm), the reflectance is lower than 25 % in the whole spectral range (400–850 nm). By changing the diameter from 200 to 500 nm, the reflectance from 500 to 850 nm does not change very much (less than 10 %). It means that if the aspect ratio is low, the reflectance of this nanostructure in the long-wavelength region is mainly influenced by the depth (*H*) and changing diameter just cause a little difference.

The anti-reflection behavior of Si-nano-patterned structures can be understood as below. First, the Si nano-patterned structures can be considered as a graded-index layer between the Si substrates and air due to the gradually changed volume ratio. The graded index layer can effectively eliminate the light reflection as revealed by Fresnel theory [20]. Second, the suppression of light reflection can also be attributed to the strong scattering between the incident light and formed nano-patterned structures because the light wavelength is comparable with the geometric size of Si nanostructures. According to Mie’s scattering theory, the strong forward scattering results in the enhanced optical absorption of incident light which can significantly reduce the surface reflection [21]. With increasing the depth of Si nano-patterned structures, the incident light can be trapped more effectively which improves the anti-reflection behaviors as shown in our simulation results. If the depth remains constant, which indicates the thickness of the graded index layer does not change. However, if the diameter of Si nano-patterned structures becomes small, the scattering between the incident light and nano-patterned structures is enhanced. Therefore, the reflectance is decreased in the whole spectral range as shown in Fig. 5. Usually, the significantly anti-reflection behaviors were usually realized in the nanostructures with high aspect ratio. Our simulation results suggest that the good anti-reflection characteristics can also be achieved by using Si nanostructures with lower aspect ratio, which is in well agreement with the previous work [17], in which, they found that the optical absorption can be obviously enhanced by forming low aspect ratio hemispherical nanopit structures. It is worth noticing that the reflectance will be strongly suppressed when the wavelength is comparable with diameter (Fig. 5*D* = 400 nm, 500 nm), and the possible reason may due to the strongest resonant scattering effect when the size is equal to the wavelength of the incident light and it is needed to be further clarified in our future work.

**Fig. 6:**
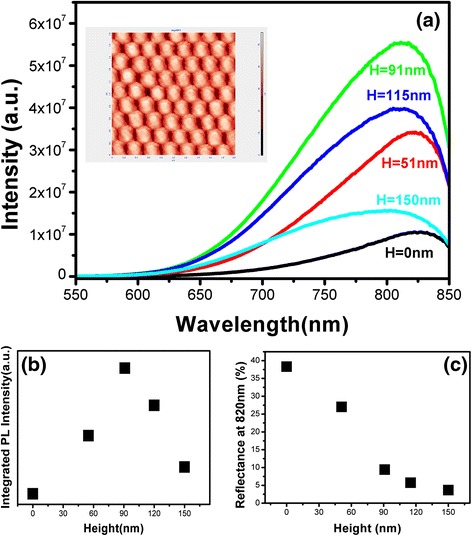
**a** Room temperature photoluminescence of Si QDs/SiO2 multilayers on flat and nano-patterned substrates under the excitation of He–Cd laser (325 nm). **b** Integrated PL intensity of samples. **c** Reflectance at 820 nm of different samples

In order to demonstrate the effect of formed Si nano-patterned structures on the device performance, we fabricate the light emission devices based on Si QDs/SiO_2_ multilayers on the nano-patterned structures with various depths. The surface morphology of samples after depositing Si QDs/SiO_2_ multilayers is checked by AFM technique. As shown in the inset of Fig. 6a, the periodic nano-patterned structures are still kept which indicates the good conformal film deposition characteristics by using the Si nanostructures with low aspect ratio.

Figure 6a shows the room temperature photoluminescence (PL) spectra from Si QDs/SiO_2_ multilayers on flat and nano-patterned structures excited by Hd–Cd laser with wavelength of 325 nm. The PL peak is centered at around 820 nm. The origin of 750–850 nm emission is still under the debate. Some groups attributed it to the quantum confinement effect, and the others believed it is due to the radiative recombination of photo-excited electron-hole pairs via interface states [22–26]. For example, Franzò et al. reported that their EL peak is due to electron-hole recombination in the Si nanocrystals and not to defects [23], but N. Daldosso et al. concluded that the PL of Si/SiO_2_ system is surface related [24]. Since we have measured the PL spectra from Si QDs-based multilayers with various dot sizes and we found that the peaks position is size independent, which is contrary to the quantum confinement expect. We attribute the 820 nm emission in our case mainly to the recombination of electron-hole pairs via the interface states on the Si nanocrystal, though we cannot completely rule out of the contribution from the band-to-band recombination within the Si QDs due to the quite broad PL band. Qin et al. also attributed the observed visible light emission to the nc-Si/SiO_2_ interfacial states based on their experimental results although the excitation of electrons and holes occurred within the Si QDs [22].

It is clearly shown that the PL intensity is obviously enhanced by using nano-patterned structures. With increasing the etching depth, the PL intensity is first enhanced and then decreased. The results are shown in Fig. 6b, and the maximum PL intensity can be obtained by using nanostructures with depth of 91 nm, which is improved by 6.6-fold compared with that of flat one. The enhanced PL intensity can be explained in terms of the improved light-extraction efficiency due to the reduction of the optical reflection on the surface of samples and interfaces between Si QDs and SiO_2_ layers by using nanostructures. As in the flat sample, the light is emitted under the all directions and only a part of light whose incident angle is less than angle of total reflection. However, the nanostructure can approximately be seen as a graded-index layer. More light can travel through a graded-index layer than that of the flat samples. The greater the *H* is, the index of this layer changes more slowly. The reflection spectra are measured and reflectance at 820 nm are given in Fig. 6c as a function of etching depth. It is found that the reflectance of Si QDs/SiO_2_ multilayers is decreased from 38.8 % for flat one to 3.6 % for sample on nano-patterned structures with depth of 150 nm. However, further increasing the etching depth causes the reduction of PL intensity, though the surface reflection becomes much lower. The possible reason is that the surface recombination will become more serious which may influence the luminescence efficiency as seen in our case. Since the thickness of SiO2 in our present work is about 2 nm and the total thickness of Si QDs/SiO2 multilayers is about 40 nm, it is possible that the part of the photo-excited carriers can tunnel through the ultrathin SiO2 layer to the surface of Si substrates, where they recombine via the surface states and result in the reduced photoluminescence intensity as we observed experimentally. It looks like that the improved device performance will be limited by the surface states by using Si nano-patterned structures which determines the optimum size parameters of nanostructures.

## Conclusions

In summary, the anti-reflection behaviors of Si nano-patterned structures prepared by nano-sphere lithography technique are studied via FDTD solutions as well as experimental observations. It is found that a good anti-reflection characteristic can be achieved by using Si nano-patterned structures even if they have the low aspect ratio which can be attributed to the graded-index as well as the strong scattering effect. With increasing the etching depth from 0 to 150 nm and reducing the diameter of nanostructures from 500 to 200 nm, the surface reflection can be obviously suppressed. In the experiment, we fabricated substrates with diameter of 300 nm and depths of 51, 91, 115, and 150 nm, and the reflectance is lower than 10 % in the measurement spectral range which is consistent with the simulation results. By depositing Si QDs/SiO_2_ multilayers on the nano-patterned structures, the reflectance can be further reduced and the reflectance at 820 nm can be as low as 3.6 %. The maximum PL intensity of quantum dots can be achieved and is improved by 6.6-folds for samples deposited on the nano-patterned structures with depth of 91 nm compared with that of flat one. However, further increasing the etching depth results in the reduction of PL intensity although the surface reflection is still reduced, which can be ascribed to the strong surface recombination of carriers. Our results demonstrate that the performance of Si-based photonic devices can be improved by optimizing the Si-based light trapping structures.
